# PKR-like ER kinase (PERK) Haplotypes Are Associated with Depressive Symptoms in People with HIV

**DOI:** 10.13188/2332-3469.1000049

**Published:** 2023-03-09

**Authors:** S Haddadi, KL Jordan-Sciutto, C Akay-Espinoza, D Grelotti, SL Letendre, B Tang, RJ Ellis

**Affiliations:** 1 Warren College, University of California, San Diego, La Jolla, CA 92093, USA; 2 Department of Pathology, School of Dental Medicine, University of Pennsylvania, Philadelphia, PA 19104, USA; 3 Department of Psychiatry, University of California, San Diego, La Jolla, CA 92093, USA; 4 Department of Medicine, University of California, San Diego, La Jolla, CA 92093, USA; 5 Department of Neurosciences, University of California, San Diego, La Jolla, CA 92093, USA

**Keywords:** Haplotypes, HIV, PKR-like ER kinase (PERK)

## Abstract

**Background:**

Depression is a debilitating and difficult-to-treat condition in people with HIV (PWH) despite viral suppression on antiretroviral therapy (ART). Depression is associated with activation of the PKR-like ER kinase (PERK) pathway, which regulates protein synthesis in response to metabolic stress. We evaluated common PERK haplotypes that influence PERK expression in relation to depressed mood in PWH.

**Methods:**

PWH from 6 research centers were enrolled in the study. Genotyping was conducted using targeted sequencing with TaqMan. The major PERK haplotypes A, B, and D were identified. Depressive symptom severity was assessed using the Beck Depression Inventory-II (BDI-II). Covariates including genetically-defined ancestry, demographics, HIV disease/treatment parameters and antidepressant treatments were assessed. Data were analyzed using multivariable regression models.

**Results:**

A total of 287 PWH with a mean (SD) age of 57.1±7.8 years were enrolled. Although the largest ethnic group was non-Hispanic white (n=129, 45.3%), African-American (n=124, 43.5%) and Hispanic (n=30, 10.5%) made up over half the sample. 20.3% were female and 96.5% were virally suppressed. Mean BDI-II was 9.6±9.5, and 28.9% scored above the cutoff for mild depression (BDI-II>13). PERK haplotype frequencies were AA57.8%, AB25.8%, AD 10.1%, and BB4.88%. PERK haplotypes were differentially represented according to genetic ancestry (p=6.84e-6). BDI-II scores were significantly higher in participants with the AB haplotype (F=4.45, p=0.0007).This finding was robust to consideration of potential confounds.

**Conclusion:**

PERK haplotypes were associated with depressed mood in PWH.Consequently, pharmacological targeting of PERK-related pathways might amelioratedepression in PWH.

## Introduction

Depression is a burdensome comorbidity in people with HIV (PWH), being 2–3 times more common in PWH than in people without HIV (PWoH), with estimates as high as 37% of PWH in a given year [1–3]. Extensive reports have delineated how depression, particularly when chronic, has multiple adverse effects including poorer medication adherence [4,5], Lower rates of viral suppression [6,7], Worse social and health-related quality of life and shorter survival [8–11]. HIV activates the unfolded protein response (UPR) [12–14], which in turn may increase the risk of depression [15]. The UPR, which is activated in animal models of depression 2957466929578616 34759791and in postmortem brain tissue from depressed individuals [15–17], is a cellular response to endoplasmic reticulum (ER) stress and protein misfolding. The protein kinase R-like ER kinase (PERK) pathway is one of the three major branches of the UPR. PERK, encoded by eukaryotic translation initiation factor 2 alpha kinase 3 (EIF2AK3), is a type I transmembrane protein kinase and stress sensor that phosphorylates eIF2*α*, which inhibits mRNA translation, thereby decreasing protein synthesis and the accumulation of misfolded proteins. The activity of the UPR system may be responsible for some of the underlying pathophysiology of depression, and this response may be involved in downstream pathways such as apoptosis, inflammation and dysfunctional cellular communication [16,18,19]. On the other hand, the relationship may be reciprocal, as inflammation is also among the stimuli that activate the PERK pathway [20]. Thus, depression and inflammation appear interrelated in PWH [21,22]. Treatment-resistant depression (TRD) in particular is associated with a heightened inflammatory response [23], and treatment with the anti-inflammatory tumor necrosis factor-alpha (TNF-*α*) antagonist, infliximab, has been shown to improve TRD.

Additionally, PERK-eIF2*α* upregulation activates the NLR family pyrin domain containing 3 (NLRP3) inflammasome to release interleukin (IL)-1*β* and modulate ER stress-related cell death [24]. A specific haplotype of PERK, haplotype B with proposed increased kinase activity [25], has been genetically associated with increased risk for the neurodegenerative disorder progressive supranuclear palsy, in which depression is a common manifestation [26,27]. Thus, haplotypes that influence the activation of PERK may carry differential vulnerability to depression due to the associated variability in inflammatory and ER stress-related pathways that are known to influence depression [28–32]. Such pathways may be particularly important in PWH since they experience persistent inflammation despite viral suppression on antiretroviral therapy (ART). Based on these considerations, we evaluated the hypothesis that common haplotypes of PERK would be associated with different degrees of depressed mood in PWH. Because of the reciprocal relationship between inflammation and PERK, we hypothesized that inflammation might serve as a mediator between PERK haplotypes and depressed mood.

## Methods

Participants underwent standardized clinical and laboratory evaluations at 6 U.S. academic centers in the CHARTER study between April 2016 and January 2020. Inclusion criteria included HIV infection and willingness to undergo the research assessments. All study procedures were approved by the Institutional Review Board (IRB), and all participants provided informed consent. Exclusion criteria were active neurological illnesses other than HIV, active psychiatric disorder (e.g., psychosis), or substance use disorder that might interfere with completing study evaluations.

### Clinical evaluations:

Depressed mood was assessed using the Beck Depression Inventory (BDI)-II including the BDI cognitive, affective, and somatic subscales8991972. Lifetime major depressive disorder (MDD) and substance use disorders were assessed using the computer-assisted Composite International Diagnostic Interview (CIDI) [33], a structured instrument widely used in psychiatric research. The CIDI classifies current and lifetime diagnoses of mood disorders and substance use disorders, as well as other mental disorders. A trained clinical examiner interviewed and examined participants to collect information such as antiretroviral treatments, nadir CD4^+^ T cell counts and current antidepressant use. Additional assessments of the clinical impact of depression included dependence in activities of daily living, employment and quality of life. Quality of life was assessed using the Medical O outcomes Study HIV Health Survey Short Form 36 (MOS-HIV SF-36) [34], a reliable and valid tool for assessing overall quality of life, daily functioning, and physical health [35,36]. The MOS-HIV contains 36 questions that assess various physical and mental dimensions of health. Items are grouped into two overall categories (Physical and Mental Health), with 11 subcategories (Physical functioning, Role functioning, Pain, Social functioning, Emotional well-being, Energy/fatigue, Cognitive functioning, General health, Health distress, Overall QoL, Health transition). These are scored as summary percentile scales ranging from 0 to 100, with higher scores indicating better health. Dependence in instrumental activities of daily living (IADLs) was assessed with a modified version of the Lawton and Brody Scale that asks participants to rate their current and best lifetime levels of independence for 13 major IADLs such as shopping, financial management, transportation, and medication management [37,38]. An employment questionnaire asked about job status, work productivity, accuracy, and quality; effort required to do one’s usual job; and fatigue with the usual workload [34].

### Clinical laboratory evaluations:

HIV infection was diagnosed using enzyme-linked immunosorbent assay with Western blot confirmation. HIV RNA in plasma was measured using commercial assays and deemed undetectable at a lower limit of quantification (LLQ) of 50 copies/mL. CD4^+^ T cells were measured by flow cytometry, and nadir CD4^+^ T cell count was assessed by self-report.

Soluble biomarkers were measured by immunoassay: soluble tumor necrosis factor receptor II (sTNFR-II), D-dimer, interleukin (IL)-6, C-reactive protein (CRP), monocyte chemoattractant protein (MCP)-1, soluble CD40 ligand (sCD40L), soluble CD14 (sCD14), and neopterin. We selected these biomarkers based on previous studies showing their link to depressed mood [32,39–43].

Genotyping was performed using TaqMan SNV genotyping assays (Life Technologies) for rs867529, rs1805165, and rs13045. The assays were performed by polymerase chain reaction as reported previously [29]. Genotypes were visualized and called using a 7900HT Fast Real-Time PCR system and the allelic discrimination function of the Sequence Detection System V.2.4 (Applied Biosystems, Waltham, MA, USA). The major PERK haplotypes A, B, and D were identified as previously described based on three single nucleotide polymorphisms (SNPs) in the EIF2AK3 gene: rs867529(Ser136Cys), rs13045(Arg166Gln), and rs1805165(Ser704Ala) forming coding haplotypes of three highly conserved residues: Haplotype A (conserved): Ser136-Arg166-Ser704; Haplotype B (divergent): Cys136-Gln166-Ala704.; Haplotype D (divergent): Ser136-Gln166-Ser704. Markers of ancestry were analyzed using EIGENSTRAT software to generate principal components [35]. Model-based clustering on the top three principal components, using the mclust R package (https://www.stat.washington.edu/mclust/), was used to assign individuals to genetic ancestry clusters [44].

### Statistical analyses:

Demographic and clinical characteristics were summarized using means and standard deviations, medians and interquartile ranges, or percentages, as appropriate. Log_10_ transformation was used to normalize the biomarker values. A factor analysis was used to reduce the dimensionality, and analysis of variance (ANOVA) was used to compare BDI-II across haplotypes. Secondary analyses evaluated correlations of BDI-II with quality of life (MOS-HIV), neurocognitive function, and employment status.We used multivariable linear regression models to test interaction effects. In the absence of an interaction, additive effects were tested. Relevant covariates including genetically-defined ancestry, demographics, HIV disease and treatment parameters, and antidepressant treatments were assessed using multivariable regression models. Analyses were conducted using JMP Pro version 15.0.0 (SAS Institute, Cary, NC, 2018).

## Results

The cross-sectional dataset included 287 PWH, including 58 (20.3%) females, with a mean age (SD) of 57.1 (7.76) and median CD4^+^ of 31.3/μL, and 276 (96.5%) PWH were virologically suppressed on ART. Self-reported race/ethnicities were African American (n=124, 43.5%), Hispanic (n=30, 10.5%), non-Hispanic white (n=129, 45.3%), other (n=2, 0.70%). Mean BDI-II was 9.6; 83 (28.9%) exceeded the cutoff for mild depression.

**Figure 1:**
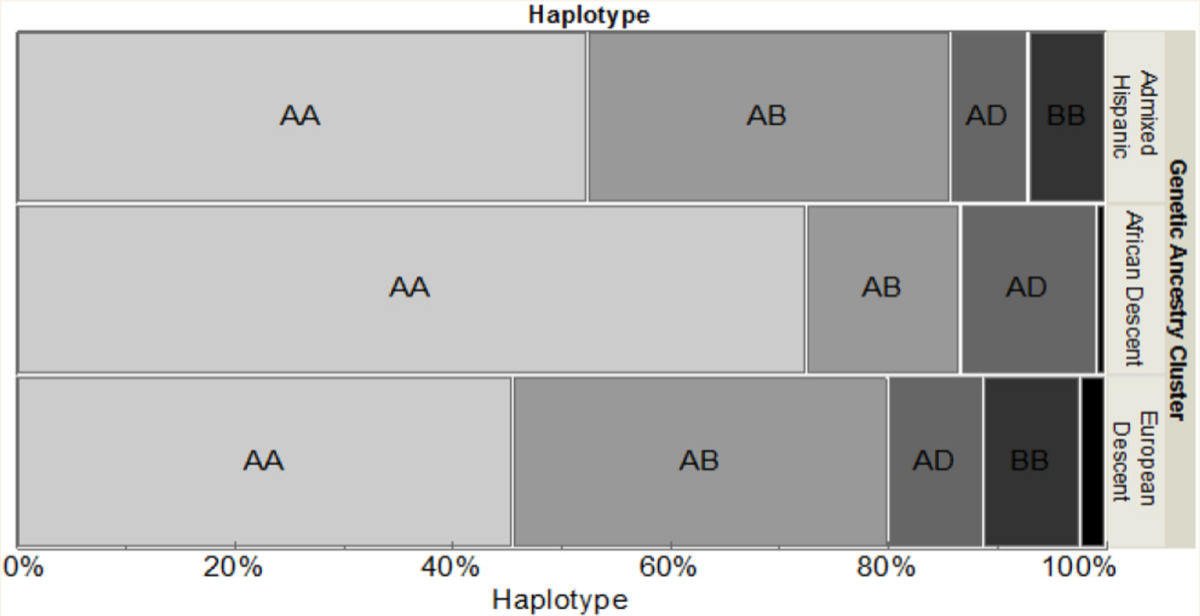
PERK haplotypeswere differentially distributed according to genetically-determined ancestry.

The distribution of haplotypes was as follows: AA57.8%, AB25.8%, AD10.1%, BB4.88%, BD1.39%. As shown in Figure 1, the haplotypes were distributed differently with respect to genetically determined ancestry. No participants of African descent harbored haplotype BB, and no participants of Hispanic descent harbored haplotype BD. The rs1805165 and rs867529 SNPs were 100% concordant across all participants. Participant demographic and clinical characteristics by haplotype are shown in Table 1.

Forty-four percent of participants had a history of meeting the criteria for MDD. Twenty-nine percent had depressed mood of at least mild severity (BDI-II score > 13). BDI-II scores were not significantly related to demographic or HIV disease characteristics (all p values >0.05). Those on antidepressant medications had worse depressed mood (BDI-II 16±10.7 versus 12.2±10.2). The distribution of haplotypes was independent of antidepressant use (p=0.252).

**Figure 2:**
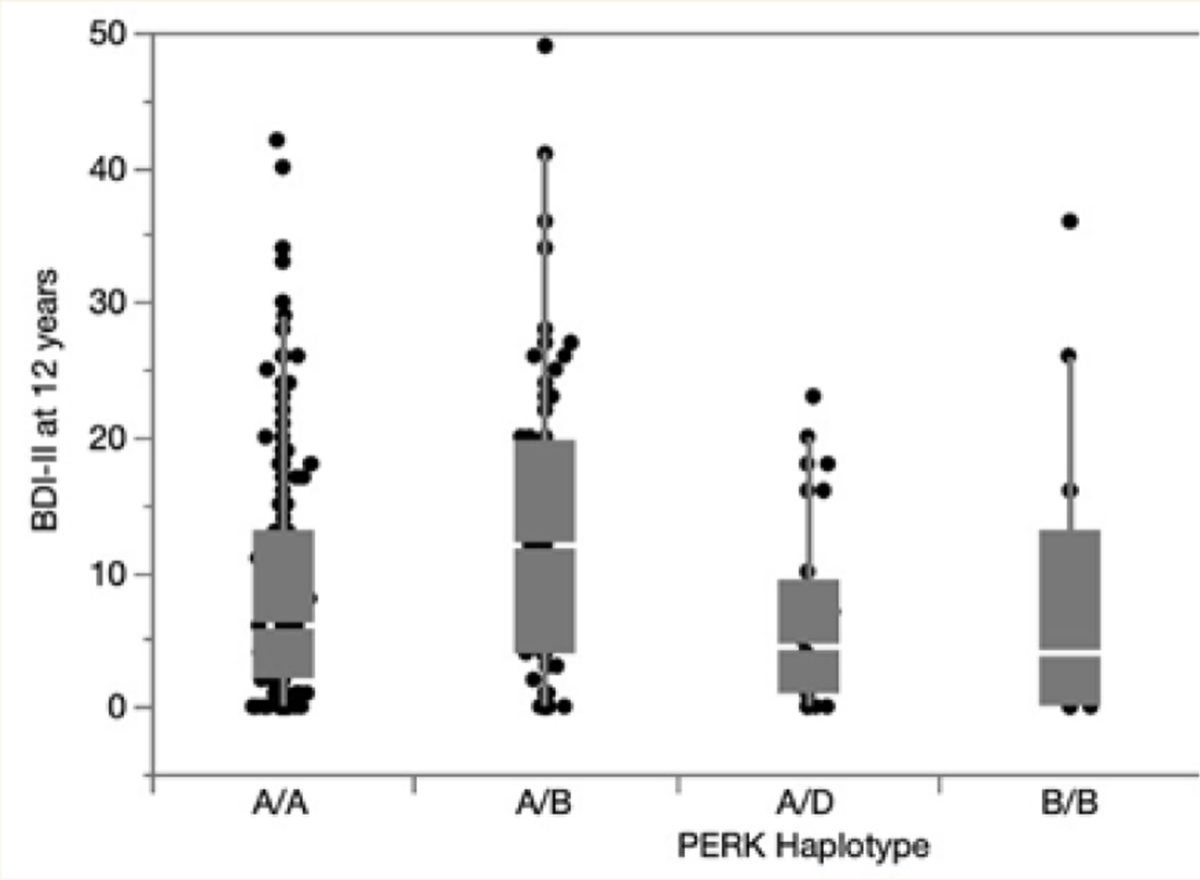
Depression scores (BDI-II; higher = worse mood) according to PERK genotype. The numbers below each box plot are the numbers of participants with each haplotype.

ANOVA demonstrated a significant overall effect of haplotype on depressive symptom severity (F=3.63, p=0.0067, (Figure 2). All BDI-II subscales contributed to the association: cognitive F=2.54, p=0.0404; somatic F=2.823, p=0.0254; affective F=2.517, p=0.0417. Follow-up pair wise comparisons among the groups using Student’s t-test showed that those with the AB haplotype had significantly worse depressive symptom severity than those with the most common AA haplotype (12.9±10.8 versus 8.83±8.83, p=0.0003) and BB (8.14±10.92, p=0.0417) haplotypes. Concordant with the results of depressive symptom severity as indexed by the BDI-II score, we found that the occurrence of incident MDD over the year before the visit was highest in those with haplotype AB (22/70, 31.4%), followed by those with haplotypes AA (43/148, 29.1%), AD (2/24, 7.69%), BB (1/13, 7.14%), and BD (0%) (p=0.0134). Similarly, PWH with the AB haplotype had the highest rate of lifetime MDD (51/74, 69.0%), compared to AA (56/165, 66.1%), AD (15/29, 51.7%), BB (6/14, 42.9%) and BD (1/3, 33.3%; p=0.0175). The haplotype–BDI-II relationship was driven by rs1805165/ rs867529. For rs1805165, heterozygotes (GT) had higher BDI-II scores (12.6±10.7) than homozygotes (GG, TT; 8.14±10.9 and 8.52±8.60; p=0.0047).

In follow-up secondary analyses, we tested the hypotheses that the different SNPs might contribute additively or synergistically to depressed mood. In a multivariable regression predicting BDI-II from rs867529 (100% concordant with rs1805165), rs13045 and their interaction, the interaction term was non-significant, while the separate main effects were significant (for rs867529, p=0.0025; for rs13045, p=0.0074; full model p=0.0067).

### Potential confounds

Since the haplotypes were distributed differently according to genetically determined ancestry, we assessed main effects of genetic ancestry on depression and the potential interaction between PERK haplotype and ancestry. In a multivariable model, the interaction term was not significant (p=0.695), and after removing it from the model, only haplotype was significant (haplotype p=0.00167; ethnicity p=0.347). Lifetime substance abuse diagnoses were significantly associated with worse depressed mood (mean±SD, 11.2±10.0 versus 7.25±8.00, p=0.0006) and with PERK haplotype (AB 81.1%, AA 78.2%, AD 55.7%, BB 64.3%, BD 100%, 0.0380). In a multivariable model, both haplotype and lifetime substance abuse diagnosis were significant (p, 0.0136 and 0.00101, respectively). Their interaction was not significant. Haplotypes were not significantly associated with current or nadir CD4 (ps=0.502, 0.442, 0.762). Viral suppression was significantly related to haplotype, being highest in haplotype BD; in a multivariable regression predicting BDI-II from viral suppression, haplotype and their interaction, viral suppression and haplotype were not significant (ps>0.05).

### Univariable association of biomarkers with BDI-II at the first visit

Concentrations of soluble biomarkers in plasma did not correlate with depressive symptoms: CRP (r=−0.00817, p=0.923), D-Dimer (r=−0.00445, p=0.958), IL-6 (r=0.08535, p=0.3091), MCP-1 (r=−0.04532, p=0.5897) and neopterin (r=0.0724, p=0.3883). Correlations were not significant for sCD14 (r=0.00396, p=0.9624), sCD40L (r=0.05, p=0.5517) and sTNFR-II (r=0.087, p=0.2968). A factor analysis was used to reduce the dimensionality of the biomarkers. The analysis yielded 3 Factors, with Factor 1 loading on sTNFRII and D-dimer, Factor 2 loading on D-dimer, IL-6 and CRP and Factor 3 loading on MCP-1 and sCD40L. None of the biomarker factors was associated with BDI-II (data not shown). Haplotypes were not significantly related to any of the biomarker factors (data not shown).

### Adverseimpact of depression on IADLs, employment, and quality of life

Worse depressed mood correlated with reduced quality of life, both physical (r=−0.560, p=4.67 × 10^−50^) and mental (r=−0.831, p=3.95× 10^−17^). Those with worse depression reported greater need for assistance in IADLs (p=3.83 × 10^−8^), and worse depression was associated with a higher risk of unemployment (p=7.3 × 10^−5^).Worse depressed mood was associated with worse memory complaints (r=0.547, p=3.88 × 10^−23^), language complaints (r=0.480, p=1.82 × 10^17^), motor complaints (r=0.371, p=1.65 × 10^−10^), sensory complaints (r=0.285, p=5.68 × 10^−29^), motor complaints (r=0.558, p=1.65 × 10^−10^),cognitive complaints (r=0.285, p=2.93 × 10^−24^) and total complaints (r=0.603, p=5.68 × 10^−29^).

## Discussion

We found that specific PERK haplotypes explained a substantial fraction of the variance in depressed mood in PWH. The effects of PERK haplotype on depressed mood were robust to consideration of genetically determined ancestry, demographics, and disease status. Worse depressed mood was associated with a severe adverse impact on quality of life, employment and IADLs. We anticipated that inflammation might mediate the significant association between PERK haplotypes and depression. However, we found instead that the effects of PERK haplotypes on depressed mood were independent of inflammation. The relatively small sample size might explain why we did not find inflammation to mediate the relationship between the haplotypes and depressed mood. Also, we did not measure some mediators that are particularly important in downstream PERK pathways, including the NLRP3-associated cytokines IL-1*β* and IL-18. Alternative interpretations of these results are that additional, unobserved variables might have influenced depressed mood or mediated the effects of PERK haplotypes on depressed mood.

Our observations are consistent with an extensive literature on the role of PERK in depression. For example, C/EBP homologous protein (CHOP) Transcription Factor and X-box-binding factor 1 (XBP1) - both downstream indicators of the PERK-mediated UPR and markers of upregulated ER stress – are elevated in PWoH with MDD [45–47]. These observations are relevant because HIV is associated with the upregulation of PERK despite viral suppression [48,49]. The implicated roles of PERK in the context of HIV infection are multipronged. HIV-induced neuroinflammation inhibits oligodendrocyte maturation via glutamate-dependent activation of PERK, and blocking PERK protects oligodendrocyte precursor cells from HIV/monocyte-derived macrophage-mediated inhibition of oligodendrocyte maturation [50]. HIV Tat-mediated induction of human brain microvascular endothelial cell apoptosis involves endoplasmic reticulum stress and mitochondrial dysfunction [51]. Furthermore, antiretroviral drugs and IL-1*β* induce the UPR, AEG-1 expression, increased intracellular calcium, and mitochondrial depolarization in astrocytes [52].

The NLRP3 inflammasome is persistently upregulated in virally suppressed PWH [53,54]. The NLRP3 gene codes for the NALP3 protein (cryopyrin), a member of the NLRP3 inflammasome complex. This complex is an intracellular sensor that detects microbial motifs and endogenous danger signals such as reactive oxygen species and lysosomal damage [55], resulting in the assembly and activation of the inflammasome [56]. This leads to caspase 1-dependent release of the pro-inflammatory cytokines IL-1*β* and IL-18, as well as to pyroptosis, a rapid, inflammatory form of lytic programmed cell death. NLRP3 remains activated in virally suppressed PWH [53,54]. Inflammaging and NLRP3 contribute specifically to neurodegenerationin HIV affecting neurotransmitter systems and neurocircuits regulating motivation, driving anhedonia [57–63]. Increased inflammatory cytokines, including those regulated by NLRP3, are regularly detected in blood and cerebrospinal fluid samples of depressed PWH [64–66]. High levels of IL-1*β* and IL-18 deplete synaptic serotonin, dopamine and norepinephrine, contributing to depression, particularly anhedonia [67]. Dopamine metabolism in the nucleus accumbens is disrupted in MDD [68]. Both IL-1*β* and IL-18 affect dendritic sprouting, synaptic plasticity, long-term potentiation, growth factors, and neurogenesis and modulate the HPA axis, affecting the stress response [69–71]. Mice exposed to unpredictable stress show inflammasome activation, IL-1β release, microglial activation and reduced hippocampal neurogenesis [72]. Treatment with iptakalim, which negatively regulates NLRP3, lowers inflammation, improves neurogenesis and benefits behavior [72]. We did not study IL-1*β* and IL-18, perhaps explaining why we did not find inflammation to be associated with PERK haplotypes and depression.

The role of PERK haplotypes in depressive mood may be leveraged for future treatment. PERK interventions using available PERK inhibitors are being explored as remedies for cellular dysfunction in chronic neurodegenerative disorders [73]. For example, one study reported that in an animal model, treatment with edaravone prevented the activation of PERK-related pathways [74]. Similarly,in preclinical models of frontotemporal dementia and prion disease [75,76], treatment with the potent and selective PERK inhibitorGSK2606414 demonstrated neuroprotective effects. Another study reported that GSK2606414 treatment prevented loss of dendritic spines and improved memory outcomes in mice after focal brain injury [77]. However, given that PERK is required for reestablishing cellular homeostasis, its inhibition may be associated with adverse effects, such as that observed in PERK knockout mice exhibiting altered glucose metabolism [78]. Another therapeutic avenue involves mitigating translation attenuation mediated by eIF2*α*, one of the targets of PERK. Indeed, several compounds targeting the modulation of eIF2*α* phosphorylation have been developed as potential therapeutics in neurodegenerative disorders and white matter disease. For example, salubrinal inhibits eIF2*α* phosphatase [79], and guanabenz and Sephin1 selectively inhibit the eIF2α phosphatase complex [80,81]. Alternatively, trans-ISRIB counteracts the eIF2α-mediated translational attenuation by interacting with eIF2B, allowing GEF activity even in the presence of p-eIF2*α* [82]. However, as a caveat to the approaches targeting eIF2*α*, PERK is one of the four kinases that can phosphorylate eIF2α; therefore, these approaches impact signaling by the other three eIF2α kinases, GCN2, IRE1a, and HRI [83]. The impact of PERK genetic variants in implementing therapeutic interventions aimed at PERK or its target eIF2*α* should be considered.

Strengths of this study include the diverse, multicenter cohort, the rigor of the depression ascertainment, the concomitant characterization of PERK haplotypes, biomarkers of inflammation and immune activation, the biomarker dimensionality reduction approach, and the breadth of characterization of impact on activities of daily living and quality of life.

Limitations of this study include the inability to assign causal roles, and the potential omission of individuals with the depressed AB haplotype and important unobserved variables. The rate of virologic suppression was low compared to modern cohorts; this may have influenced the prevalence of depression, or vice-versa. Females were underrepresented here, so the results may not be generalizable to them. We studied only individuals with HIV infection; it is possible that PERK genetic variations also associate with depression in people without HIV or in other neurodegenerative diseases where there is evidence that the unfolded protein response is activated [84,85].

## Conclusion

*Aspergillus* species were the most common fungi isolated from the indoor environment while *Trichophyton* species were also isolated from the plant soil surface. A high incidence of fungi was seen in the indoor environment of residents suffering from allergies and asthma. Many therapeutic options are effective against allergic rhinitis, including a combination of antihistamines, corticosteroids (intranasal and oral), and anti-leukotrienes. The treatment efficiency was improved with hygienic environmental conditions by avoiding fungal contaminants which were the major trigger in indoor environments.

## Figures and Tables

**Table 1:** Participant demographic and clinical characteristics by haplotype.

Haplotype						
	AA	AB	AD	BB	BD	p
N	166	74	29	14	4	
Age in years, mean (SD)	57.0±7.76	56.6±7.61	58.6±6.76	56.0±10.8	62.0±3.92	0.513
Sex female, N (%)	35 (21.2%)	15 (20.3%)	8(27.6%)	0 (0%)	0 (0%)	0.275
Self-reported race/ethnicity, N (%)						
African origin	89 (53.9%)	19 (25.7%)	16 (55.2%)	0	0	
Admixed Hispanic	17 (10.3%)	9 (12.2%)	2 (6.90%)	2 (14.3%)	0	6.84e-6
European	58 (35.2%)	46 (62.2%)	10 (34.5%)	12 (85.7%)	3 (100%)	
Other	1 (0.61%)	0	1 (3.45%)	0	0	
HIV duration, median (IQR)	22.9(17.2, 28.4)	21.7(16.7, 29.1)	23.5(16.2, 26.7)	22.6(15.7, 28.4)	22.3(20.8, 29.9)	0.978
Current CD4^+^*, median (IQR)	32 (24.3, 41.3)	29 (21, 42)	29 (26, 38)	24 (15.5, 39.5)	27(20.6, 33.5)	0.500
Nadir CD4^+^*, median (IQR)	119 (20.8, 240)	102 (20.8, 227.8)	115 (30, 231)	71.5 (9.3, 188.8)	8 (0, 49)	0.442
On ART, N (%)	160(96.4%)	72(97.3%)	27 (93.1%)	14 (100%)	3 (100%)	0.860
Undetectable plasma HIV RNA on ART, N (%)	108 (85.7%)	54 (84.4%)	17(73.9%)	9 (64.3%)	3 (100%)	0.0467

* T-lymphocytes/*u*L
